# The role of economic empowerment for mental health in the Global South

**DOI:** 10.1192/bjo.2025.10804

**Published:** 2025-09-22

**Authors:** Monika Müller, Soumitra Pathare, Abhijit Nadkarni

**Affiliations:** Faculty of Health Sciences and Medicine, University of Lucerne, Switzerland; Lucerne Psychiatric Services, Lucerne, Switzerland; Centre for Mental Health Law and Policy, Indian Law Society, Pune, Maharashtra, India; Centre for Global Mental Health, Department of Population Health, London School of Hygiene and Tropical Medicine, London, UK; Addictions and Related Research Group, Sangath, Porvorim, Goa, India

**Keywords:** Low- and middle-income, mental health services, economic empowerment, livestock transfer, cash plus programmes

## Abstract

There is a great potential for carefully designed economic empowerment programmes to improve mental health in recipients and their significant others. Onono and colleagues interviewed 62 caregiver-adolescent dyads on the effect of an economic empowerment intervention consisting of microcredits to purchase farming implements and a water pump to irrigate crops throughout the year combined with agricultural and financial training. Their intersectoral economic empowerment intervention decreased parental stress, parental absenteeism as well as harsh parenting and disciplining practices. This translated to better caregiver-adolescent communication and improved household dynamics, thus increasing the psychological well-being of adolescents. The research contributes to a growing evidence base on the importance of economic empowerment interventions for mental health by generating hypotheses on mechanisms of action.

**Figure:**
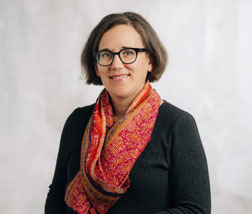

Globally, one in ten people live in extreme poverty, defined by the World Bank as living on less than 1.90 USD per day.^1^ This translates to 738 million people living in extreme poverty, 74% of them in the global South. The complex and bidirectional relationship between poverty and mental health is well documented.^2^ It is of particular importance in the Global South where poverty rates are high and financial resources volatile.

Economic empowerment allows people who live in poverty to think beyond immediate daily survival and to exercise greater control over their resources and life choices. It enables households to make investments in health and education and it gives financial security to afford vital necessities without having to sell assets or incur debt. Economic empowerment programmes commonly include cash transfers, microcredit, and livestock or other productive asset transfers. Cash transfers began in the 1990s as a response to the negative impacts of the 1980s debt crisis in Latin America with the idea of allowing designated beneficiaries to meet their basic needs. They have become widespread in the Global South, particularly since the early 2000s.^3^ Between 2007 and 2010, development assistance spending on cash transfers increased substantially from USD 23 million to USD 150 million.^4^ Microcredit programmes, the second most common economic empowerment intervention, entail providing small loans on easy terms to people who live in poverty and who are thus ineligible for traditional financial services due to their insolvency. Following the 1974 famine in Bangladesh, Nobel laureate Muhammad Younus in collaboration with Grameen Bank began to offer such loans to people who wanted to start their own small businesses. Currently, hundreds of similar programmes exist in Asia and Sub-Saharan Africa. Finally, productive-asset transfers provide livestock or other assets to households and are typically combined with training on how to utilise them for production.^5^ They are viewed as a promising way to transform the economic lives of landless, assetless, agricultural labourers.^5^

Latest developments in the field are intersectoral economic empowerment programmes that combine the financial component with health and educational interventions.^6^ Intersectoral approaches typically show more health benefits than programmes delivered within firm sectoral boundaries because the ‘plus’ component ensures the necessary impact of the financial component. The *Shamba Maisha* intervention which was developed and evaluated with a randomised controlled trial in rural Kenya is one such intersectoral economic empowerment programme.^7^ Participants randomised to the intervention arm received a financial component consisting of microcredits to purchase farming implements and a water pump alongside agricultural and financial training as the educational component. The pump allowed them to irrigate crops throughout the year, avoiding seasonal crop failures.

The article by Onono and colleagues presented in this issue was a qualitative study nested in the *Shamba Maisha* trial.^7^ The aim of the study was to explore how the economic empowerment intervention influenced parenting practices, caregiver-adolescent relationships and, consequently, the psychosocial well-being of adolescents. The researchers interviewed 62 caregiver-adolescent dyads and found that *Shamba Maisha* may have led to decreased parental stress, decreased parental absenteeism as well as reduced harsh parenting and disciplining practices. The authors hypothesised that this translated to better caregiver-adolescent communication and improved household dynamics, thereby increasing the psychological well-being of adolescents. This research contributes to a growing evidence base on the importance of economic empowerment interventions for mental health by generating hypotheses on mechanisms of action. Their qualitative data suggest two main pathways through which economic empowerment programmes at the household level may lead to a positive effect on adolescents’ mental health. First, the researchers hypothesised that decreased parental and adolescent stress might improve the mental health of adolescents. Other studies based on quantitative and biological data support this potential mechanism of action. For example, a study conducted in 1440 households in Kenya showed that individuals living in households that received cash transfers had significantly lower levels of blood cortisol compared with those living in households not receiving cash transfers.^8^ Similarly, cash transfer programmes in Mexico and South Africa found lower levels of stress and depression (measured using psychometric tools) in children of households that received cash, with a particularly strong effect in children living with a parent having depression.^9,10^ Second, Onono and colleagues found that parents in households receiving the economic empowerment intervention reported not only having more time with their children but also being emotionally present when they were with their children as opposed to long working hours to obtain the necessary resources for the family. Parental absenteeism and, consequently, lack of parental monitoring is hypothesised to lead to the risky behaviour of adolescents. Previous research supports these results.^11^

The main limitation of the featured qualitative study is linked to the timepoint of data collection and analysis, which took place at the end of study participation in the main trial.^7^ The semi-structured interviews were performed approximately one year after having received the intervention and thus stress levels, household dynamics and absenteeism during this past period of intervention implementation may be subject to recall bias. Additionally, social desirability bias is likely to play a more important role after having received the intervention. Participants interviewed in the intervention arm might have overemphasised the benefits.

Based on the work of Onono and colleagues we suggest several areas for further research. First, future research should explore additional mechanisms of action on how economic empowerment programmes improve mental health. Preliminary evidence suggests neurocognition as an important mediator because poverty interferes with executive functions and decision-making processes.^12^ To make choices while living in poverty requires great mental effort because different options with financial implications need to be carefully weighed against each other, with little margin for error. This increased mental load lowers self-control mechanisms which are important to postpone gratification. For example, the attentional performance of Indian farmers depended on the harvesting period, with reduced attentional capacity before harvest when the farmers typically face financial constraints.^13^ However, studies investigating the role of neurocognition that are nested in trials evaluating economic empowerment interventions are yet to be realised. Second, the work of Onono and colleagues highlights the importance of including significant others in future research on economic empowerment and mental health. Previous research reports increased stress, anxiety and depression in the spouses of people living with depression.^14^ Additionally, mental health problems contribute to household poverty. This is of particular importance in the Global South where governmental financial risk protection measures are lacking. Reduced income resulting from depression, associated disability or unemployment and high out-of-pocket health care expenditures place people with depression, and their families, at risk of impoverishment.^15^ Therefore, people with mental illnesses and their families who live in the Global South define a particularly vulnerable population in need of innovative solutions that target both their mental health problem as well as their fragile economic situation. This suggests a third area of future research which is to design and evaluate intersectoral economic empowerment interventions for people with mental illness. Previous trials such as the *Shamba Maisha* trial^7^ included people from the general population without mental illness and evaluated the effect on mental health as secondary outcomes, often using unvalidated tools to measure the mental health outcomes.

In summary, there is a great potential for carefully designed economic empowerment programmes to improve mental health in recipients and their significant others, especially if a strong economic empowerment component is combined with an evidence-based mental health intervention. This will simultaneously address mental health and poverty as a key social determinant of poor mental health and thus break this two-way relationship. Intersectoral economic empowerment programmes like the *Shamba Maisha* have the potential to integrate mental health into the social welfare sector in the Global South. This has several advantages because such programmes are likely to inform both health and economic policy. This is of importance given the fact that mental health has not been considered a priority by economists even though economic policies can improve mental health. Furthermore, it will indirectly open another route to increase access to mental health care substantially. Intersectoral solutions are more likely to lead to Universal Health Care coverage which lies at the core of the third Sustainable Development Goal as compared to exclusively financial interventions that are delivered within firm sectoral boundaries.

## Data Availability

Data availability is not applicable to this article as no new data were created or analysed in this study.
